# Reformation of science publishing: the Stockholm Declaration

**DOI:** 10.1098/rsos.251805

**Published:** 2025-11-05

**Authors:** Bernhard Sabel, Dan Larhammar

**Affiliations:** ^1^ Department of Medical Psychology and Department of Computer Science, Otto-von-Guericke University of Magdeburg, Magdeburg, Sachsen-Anhalt, Germany; ^2^ Department of Medical Cell Biology, Uppsala University, Uppsala, Sweden

**Keywords:** fake publications, science integrity, fraud, paper mill, artificial intelligence, AI, libraries, scholarly academies

## Abstract

Science relies on integrity and trustworthiness. But scientists under career pressure are lured to purchase fake publications from ‘paper mills’ that use AI-generated data, text and image fabrication. The number of low-quality or fraudulent publications is rising to hundreds of thousands per year, which—if unchecked—will damage the scientific and economic progress of our societies. The result is editor and reviewer fatigue, irreproducible experiments, misguided experiments, disinformation and escalating costs that devour funding from taxpayers intended for research. It is high time to reevaluate current publishing models and outline a global plan to stop this unhealthy development. A conference was therefore organized by the Royal Swedish Academy of Sciences to draft an action plan with specific recommendations, as follows. (i) Academia should resume control of publishing using non-profit publishing models (e.g. diamond open-access). (ii) Adjust incentive systems to merit quality, not quantity, in a reputation economy where the gaming of publication numbers and citation metrics distorts the perception of academic excellence. (iii) Implement mechanisms to prevent and detect fake publications and fraud which are independent of publishers. (iv) Draft and implement legislations, regulations and policies to increase publishing quality and integrity. This is a call to action for universities, academies, science organizations and funders to unite and join this effort.

## 1. A call to action

The integrity of academic publishing, a cornerstone of science,^1^ is critical for the advancement of health care, technological development and economic growth. Yet, the publishing system is targeted by three major threats: for-profit publishers create barriers to research dissemination and demand substantial fees for open access (OA) which contribute to their 25–35% profits from academia, predatory journals publish a flood of low-quality papers without adequate peer review and, most recently, ‘paper mills’ increasingly pollute the scientific literature with fake articles reporting fake data. Although numerous statements on the code of conduct for research integrity and academic publishing have been disseminated to ascertain high research quality and integrity (see table 1), low-quality and fraudulent publications have dramatically increased [1]. This upsurge is driven by incentives of a reputation economy where quantitative metrics (publication number, impact factors, h-index) often count more than the quality of the research. These metrics are gamed, among others, by the hundreds of thousands of fake publications [2,3] produced each year by a corrupt ‘paper mill’ industry [4] that sells authorships to scientists under pressure to publish and bribes editors to publish these papers [5], a development which is accelerated by AI. This situation drains financial resources provided by taxpayers and funding agencies, distorts studies, wastes efforts to perform studies that build upon them, leads to meaningless replication studies, spreads false information that is later hard to debunk, and undermines practical applications including medical care and engineering. It is arguably the largest science crisis of all time, threatening to erode people’s trust in research.

**Table 1.** Declarations for reforming scientific publishing.


**San Francisco Declaration on Research Assessment (DORA; 2012**) —**Recommendation**: Stop using journal impact factors as a proxy for research quality, evaluate research on its own merits; prioritize content over publication venue. —https://sfdora.org/
**Leiden Manifesto (2015**) —**Recommendation**: Responsible use of metrics in research evaluation. —https://www.leidenmanifesto.org/
**Fair Open Access Alliance (FOAA; 2015**) —**Recommendation:** Transition of journals from commercial to scholar-led, community-owned, not-for-profit publishing. Authors retain copyright. Transparent pricing and governance. —https://www.tu.berlin/en/ub/research-publishing/publishing/financing-for-open-access/fair-open-access
**Jussieu Call for Open Science and Bibliodiversity (2017**) —**Recommendation**: Support diverse publishing models, including small, scholar-led publishers, promoting non-profit, multilingual and community-driven approaches to publishing. —https://jussieucall.org/
**Plan S (2018**) —**Recommendation**: From 2021, all publications funded by public grants must be fully open access. Authors should retain copyright and use Creative Commons licences. —https://www.coalition-s.org/
**Helsinki Initiative on Multilingualism in Scholarly Communication (2019**) —**Recommendation**: Open access, academic autonomy in choosing language and publishing models. —https://www.helsinki-initiative.org/en
**Scholar-Led Plus (2021**) —**Recommendation**: Strengthen infrastructures and policies for scholar-led publishing. Reclaim publishing from commercial control, emphasizing equity and autonomy. —https://www.hiig.de/en/project/scholar-led-plus/
**All European Academies (ALLEA; 2023**) —**Recommendation:** Provides framework for self-regulation across all scientific and scholarly disciplines and for all research settings. —https://allea.org/portfolio-item/european-code-of-conduct-2023/

The Stockholm Declaration is a call to action for all stakeholders in science and technology organizations around the world to unite in reforming the structure of the current science publishing culture [6] and to assure academic freedom and trustworthiness by community control. Other initiatives have formulated recommendations which are compatible with the present Stockholm Declaration (see table 1). They address topics such as transparency, fairness and academic control in publishing. Most also demand shifting control of scholarly publishing away from profit-driven corporations and back to the academic community.

We urge learned societies, funding agencies, policy makers, publishers and those who translate scientific progress into practical applications to act. Trust in science is critical for informed, balanced and reliable decision-making and to preserve our global knowledge base, i.e. the scientific record, and the integrity of science itself.

Therefore, the undersigned (see §7) declare the following set of principles and call for actions of *good publishing practice* to be implemented worldwide.

1. **Academia resumes control of publishing**
  *Action*: Convert for-profit to sustainable non-profit publishing models, where the academic community owns the journals' titles and authors retain the copyright, e.g. using open access models.
  *Responsible*: Editors, libraries, scholarly academies and other scientific organizations, with the support of funders.
2. **Incentive systems to merit quality, not quantity**
  *Action*: End incentives for mass production of low-quality articles and the use of citation metrics in a ‘publish or perish’ culture of a reputation economy; focus on quality, not quantity, in hiring, tenure and funding decisions.
  *Responsible*: Members of committees who decide on hiring, promotion and grants at universities, research or funding organizations.
3. **Independent fraud detection and prevention**
  *Action*: Detect, sanction and register publishing corruption and ‘fraud tag’ fake articles, paper mills that produce fake articles and journals that publish them. Use validated quality/integrity markers to detect fake articles.
  *Responsible*: Sleuths and fraud-monitoring organizations that are independent, i.e. not funded by publishers.
4. **Legislation and policies to protect science quality and integrity**
  *Action*: Draft and implement legislation and regulations (checks and balances) to detect and penalize individual and industry-scale fraud, such as fake publications by paper mills, to protect academic integrity.
  *Responsible*: National and international legislative bodies, governments and funders.

If successfully implemented, these actions will return control of publishing to academia, reduce the soaring number of low-quality publications and fraud, minimize costs while increasing trustworthiness of science and enhance academic freedom. Otherwise, science will be increasingly undermined by profit-driven motives. In what follows, we detail these *good publishing principles and practices*.

## 2. Academia resumes control of publishing

To simplify the following discussion, we distinguish between ‘commercial publishers’ (CPs) and ‘academic publishers’ (APs). Whereas CPs advance their surplus (profit) to shareholders and private investors, APs are typically researcher-led organizations such as universities, research institutions or learned/disciplinary societies that are non-profit because they return any financial surplus to science.

- To resume control of academic publishing and increase quality of science publishing, we call on science funders (e.g. governments, foundations) to shift their funding of open access publications from CPs with unreasonable or non-transparent article processing charges (APCs) to (non-profit) researcher-led APs. This helps avoid the ongoing, uncontrolled, commercial growth of low-quality and predatory publishing, protects integrity, and significantly reduces public spending and commercial bias. It will help shift control of science publishing to the academic community,^2^ as confirmed by several best practice examples: the European Commission journal *Open Research Europe* (scientists funded by Horizon Europe Programme pay no open access fees), the IAP report,^3^ Latindex^4^ and the declaration of Mexico.^5^
- Though traditional subscription journals (hard copy or digital) are expected to continue for some time, we recommend a transition from subscriptions and transformative agreements to innovative digital open access models (free submission, free reading). However, we remain open to any other CP or AP publishing model if they adhere to the following principles:
  - Open by design (open to read, open to publish meaningful contributions).
  - Maintains ultimate human control of peer review (no peer review by AI, publisher employees or ‘editors-in-residence’).
  - Uses technologies and open-source information repositories that are interoperable.
  - Retains all rights for researchers, including AI licensing.
  - Transparently shows how the publisher is funded.
- Funders shall focus on supporting non-profit publishers, especially open-access journals, including new APs up to the point of balanced economy.
- Encourage, test and support innovative, non-profit publishing models as inspired by existing models,^6^ such as:
  - Community-reviewed preprints (e.g. *eLife* or https://peercommunityin.org).
  - ‘Living documents’ (they are flexible and can be updated continually to reflect new information).
  - Modular publishing platforms for research communities (e.g. Research Equals).
  - Preprint publishing with commenting features (like bioRxiv or medRxiv).
- Digital archiving of past and future publications should be hosted by repositories which are independent of publishing organizations, comprising a network of mirrored server hubs around the world (the ‘permanent scientific record’) to enable the ‘science-of-science’.
- The responsibility of every publisher—and their respective infrastructure, distribution models and marketing activities—shall include the following:
  - Monitor and prevent the processing of fraudulent scientific information.
  - Offer open access to all digital publication records for AI analysis by qualified scientists and transfer already published research articles to science-controlled archives.
  - Offer reasonable subscription rates and/or article processing charges (APCs) [7].
- AI use in scientific publishing and data tracking needs guidelines, but because the AI field is currently developing very fast without proper regulation, opportunities and risks will need continuous monitoring and adjustment.

## 3. Incentive systems to merit quality, not quantity

- Reward scientific achievement based primarily on quality—not quantity measures of today’s ‘reputation economy’ metric system [8].
- Discourage ‘salami-slicing’ of results to obtain ‘least publishable units’; reduce the abundance of review articles (meta-analyses) and commentaries that over-burden reviewers and can too easily be generated using AI.
- Rankings of academic achievement with metrics such as citation counts, ‘journal impact factor’ (JIF) and ‘publication number’ must be used responsibly because they often do not correlate with research quality [9] (see also CoARA—Reforming Research Assessment). ^7^
- Select appropriate achievement criteria that are compatible with respective disciplines and publishing traditions.
- Because peer review adds to researcher workload, the science community should reflect on how to incentivize it properly (e.g. tracking scientists' number of review assignments and their quality).
- Applications for funding or positions/promotions should disregard metrics and limit the number of publications to be evaluated (e.g. 10) depending on career age, academic field, etc.

## 4. Independent fraud detection and prevention

- Raise awareness of the rising industry-like fake-paper production by paper mills and offer integrity education to students, researchers and editors, as well as publishers, administrators, decision-makers and journalists.
- Develop automated fraud detection systems to identify fake publications and their producers. To avoid conflicts of interest, such quality monitoring systems should be operated by independent, researcher-controlled and certified non-profit organizations that are not under the control of or financed by for-profit publishers.
- Monitor publishing fraud such as fabrication, falsification and plagiarism (e.g. text, images, data), authorship piracy and trading, fake reviews, citation gaming, undeclared parallel manuscript submissions or other unethical behaviours [10]. Establish integrity registries with quality markers (white-listing) of institutions, editors, journals and publishers.
- Reward sleuthing, but beware of unfair ‘whistle-blowers’.

## 5. Legislation and policies to protect science quality and integrity

- Develop a consensus on how to sanction institutions that artificially bolster high publication numbers and those that protect fraudsters.
- Advise/educate and fund institutional review boards on how to ascertain publishing quality by implementing appropriate incentive systems and how to identify—and respond to—integrity and ethics violations.^8^
- Draft legal definitions of ‘fraudulent publishing’ (is it breach of contract or a crime?).
- Prohibit the use of ‘reputation economy’ scoreboards that use manipulated achievement metrics (e.g. of the impact factor or h-index^9^).
- Draft national regulations to define, monitor and sanction violations of research integrity by paper mills, authors who pay paper mills, editors and journals accepting bribes from paper mills and publishers at scale by researcher-controlled ‘research integrity bodies’, including mandatory fraud listing by indexing services.

## 6. Disclaimer

**Figure 1.**
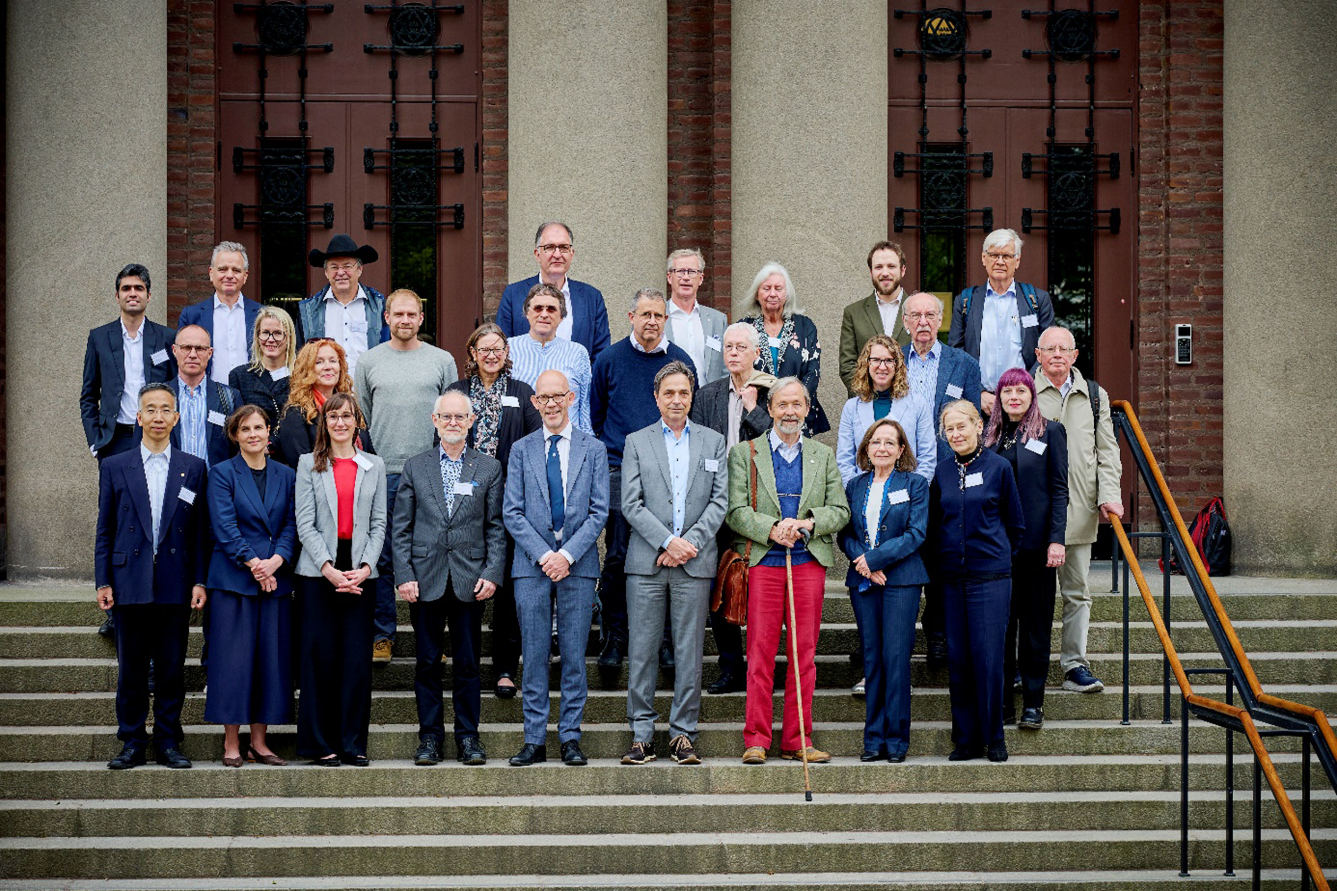
Members of the June 2025 workshop at the Royal Swedish Academy of Sciences. At the centre of the front row in a blue suit is Hans Ellegren, secretary general of the Academy; to his right is Dan Larhammar (former president of the Academy) and to his left is Bernhard Sabel. Photo: Patrik Lundin/The Royal Swedish Academy of Sciences.

This declaration was inspired by a conference held at the Royal Swedish Academy of Sciences in Stockholm, Sweden, on 9–10 June 2025 (figure 1). Conference participants from diverse disciplines, organizations and countries contributed their personal expertise to the discussions as individuals. They did not necessarily agree with every statement of this declaration, nor did they officially represent the views of their respective institution or organization. This declaration was drafted to inspire the science community and its funders to fundamentally reform academic publishing. We recognize that individual science disciplines, traditions, academies and countries may have additional needs not addressed here. Note that at this point, our declaration does not imply consent by any organization (scholarly organization, academy or publisher). However, those in support are invited to co-sign the Stockholm Declaration and become members of a global coalition of the willing, dedicated to the integrity of science publishing.

## 7. Stockholm Declaration conference participants

List of experts who undersigned the declaration.

- **International experts:**
  Claudio Aspesi (Switzerland), independent consultant; marketing Analyst.
  Boris Barbour (France), board Member, PubPeer.
  Christophe Bernard (France), chief editor, eNeuro; director of research exceptional class, INSERM, Centre national à la recherche scientifique (CNRS).
  Elisabeth Bik (USA), independent consultant and Sleuth, Harbers Bik LLC.
  Sonja Bjelobaba (Sweden), senior Lecturer, Centre for Research Ethics & Bioethics (CRB), Uppsala University.
  Geoffrey Boulton (UK), board member, International Science Council (ISC); Regius Professor Emeritus of Geology, University of Edinburgh.
  Ana María Cetto (Mexico), President, LATINDEX; Instituto de Física, Universidad Nacional Autónoma de México (UNAM).
  Marie Farge (France), director of research emeritus, INSMI, Centre national à la recherche scientifique (CNRS); member, Committee on Publishing of the International Mathematical Union (IMU).
  Gerd Gigerenzer (Germany), vice-president, European Research Council (ERC); director emeritus, Max Planck Institute for Human Development, Max Planck Society
  Ahmar Hussain (Germany), research Associate, Otto-von-Guericke University of Magdeburg.
  Jianbin Jin (China), board member, International Association of University Libraries; library Director, Tsinghua University.
  Lauren Kmec (USA), deputy executive Editor, American Association for the Advancement of Science (AAAS).
  Dan Larhammar (Sweden), University of Uppsala; past-President of the Royal Swedish Academy of Sciences.
  Bernhard Sabel (Germany), Otto-von-Guericke University of Magdeburg; state-chapter chair of German University Association (DHV); founder, Sciii gGmbH (a Science and Innovation Integrity Foundation).
  Peter Seeberger (Germany), vice-president, Deutsche Forschungsgemeinschaft (DFG); director, Max Planck Institute of Colloids & Interfaces; senate member of the Max Planck Society.
  Roland Seifert (Germany), chief Editor, Naunyn-Schmiedebergs Archives of Pharmacology Pharmacol; director, Institute of Pharmacology & Research Core Unit Metabolomics, Medizinische Hochschule Hannover.
  Jennifer Trueblood (USA), Ruth N. Halls Professor of Cognitive Science and Psychological & Brain Sciences; Director, Cognitive Science Program, Indiana University Bloomington.

- **Members of the Royal Swedish Academy of Sciences (KVA**)
  Anna Dreber Almenberg (Sweden), Dept. Economics, Stockholm School of Economics.
  Lars Hultman (Sweden), Dept. Thin Film Physics, Linköping University.
  Mats Larsson (Sweden), Dept. Physics, Stockholm University.
  Nils-Göran Larsson (Sweden), former Director, Max Planck Institute for Biology of Ageing, Max Planck Society, Karolinska Institute.
  Peter Pagin (Sweden), Dept. Philosophy, Stockholm University.
  Ilona Riipinen (Sweden), Director, Bohlin Centre of Climate Research, Dept. Environmental Science, Stockholm University.
  Britt-Marie Sjöberg (Sweden), Dept. Molecular Biology, Stockholm University.
- **External consultants**
  Rod Cookson (UK), Representative, Royal Society of London.
  Xiwen Liu (China), general Director, National Science Library of the Chinese Academy of Sciences.
  Diethard Tautz (Germany), German Academy of Sciences—Leopoldina.

## 8. Co-signing opportunity

The Stockholm Declaration is also available online with a registry, where any academy, scholarly organization, funding or administrative body from any country around the world can sign up to express support of its mission—in full or in part (https://sciii-it.org/stockholm-declaration).

## Data Availability

This article has no additional data.

## Footnotes

^1^
The term ‘science’ should be understood broadly as including all disciplines, with humanities and social sciences.

^2^
https://op.europa.eu/en/publication-detail/-/publication/3603e219-6a65-11ef-a8ba-01aa75ed71a1/language-en.

^3^
https://www.interacademies.org/publication/iap-announces-publication-its-2022-annual-report.

^4^
https://en.wikipedia.org/wiki/Latindex.

^5^
https://www.issn.org/newsletter_issn/declaration-of-mexico-in-favor-the-latin-american-non-commercial-open-access-ecosystem/.

^6^
https://www.leopoldina.org/en/publications/detailview/publication/a-new-concept-for-the-direct-funding-and-evaluation-of-scientific-journals-2025/.

^7^
https://coara.eu/agreement/the-agreement-full-text/.

^8^
https://www.researchprofessionalnews.com/rr-news-europe-universities-2024-4-commission-official-help-us-change-culture-of-research-ethics/.

^9^
https://reeserichardson.blog/2024/07/18/engineering-the-worlds-highest-cited-cat-larry.
